# Low-Density Lipoproteins Induce a Pro-Inflammatory, Chemotactic Mox-like Phenotype in THP-1-Derived Human Macrophages

**DOI:** 10.3390/cells15010055

**Published:** 2025-12-28

**Authors:** Heng Yu, Radhika R. Josi, Ankur Khanna, Damir B. Khismatullin

**Affiliations:** Department of Biomedical Engineering, Tulane University, 6823 St. Charles Avenue, New Orleans, LA 70118, USA

**Keywords:** macrophage, mast cell, low-density lipoproteins, atherogenesis, TGF-β, IL-6

## Abstract

Murine macrophages exposed to oxidized low-density lipoprotein (oxLDL) polarize into a distinct Mox phenotype characterized by impaired phagocytic and chemotactic function. Although implicated in atherosclerosis, this phenotype has not been confirmed in human macrophages. Drawing parallels to human tumor-associated macrophages, and in contrast to the murine cell response, we hypothesize that LDL/oxLDL induces a hybrid Mox-like state in human macrophages, marked by the simultaneous secretion of pro-inflammatory cytokines and anti-inflammatory factors, potentially exacerbating vascular inflammation and atherogenesis. To test this, THP-1 human monocytes were differentiated into resting macrophages, then polarized into M1-like and M2-like phenotypes, followed by treatment with native LDL, oxLDL, IL-6, or their combinations. ELISA results showed that oxLDL or LDL with IL-6 polarized resting and M1-like macrophages into a Mox-like phenotype that secreted TNF-α and TGF-β1 at levels comparable to M1- and M2-like cells, respectively. The pro-inflammatory nature of Mox-like macrophages was supported by increased THP-1 adhesion to vascular endothelial cells exposed to the macrophage-conditioned media. In microfluidic assays, LUVA human mast cells migrated toward media from Mox-like macrophages, indicating enhanced chemotaxis. In summary, the pro-inflammatory Mox-like state is triggered in human macrophages by oxLDL or LDL combined with IL-6, a key regulator of the inflammatory acute-phase response. Unlike in murine cells, this state is marked by high chemotactic activity driven by TGF-β1 secretion, which promotes mast cell recruitment and contributes to atherosclerotic plaque development and Alzheimer’s disease.

## 1. Introduction

Monocyte-derived and tissue-resident macrophages (MΦs) are key regulators of inflammation, capable of orchestrating both its initiation and resolution. To carry out these roles, MΦs transition from a resting state to either a pro-inflammatory or anti-inflammatory phenotype. Dysregulation of this polarization process contributes to various pathophysiological conditions, including atherosclerosis [1]. Much of our understanding of MΦ polarization, including the classification into pro-inflammatory M1 and anti-inflammatory M2 states, has been derived from studies using murine models, particularly mouse bone marrow-derived MΦs (mBMDMs) [2,3]. These MΦs exhibit distinct and largely non-overlapping responses to chemical cues, polarizing toward the M1 phenotype in response to lipopolysaccharide (LPS) and interferon-gamma (IFN-γ), or toward the M2 phenotype when stimulated with interleukin-4 and interleukin-13 (IL-4 and IL-13) [4]. Upon exposure to oxidized phospholipids, key constituents of oxidized low-density lipoprotein (oxLDL) particles, mBMDMs adopt a dysfunctional Mox phenotype, marked by diminished phagocytic and chemotactic activity [5]. In murine aortic atherosclerotic lesions, 34% of MΦs were found to exhibit the Mox phenotype, characterized by distinct expression of heme oxygenase-1 (Hmox1), and showed less than 12% phenotypic overlap with M1 or M2 MΦs [5].

In vitro studies with human MΦs, derived from peripheral blood mononuclear cells (PBMCs) and THP-1 monocytic cells, a well-established and extensively used model to study functions and activities of human monocytes and MΦs [6], similarly indicate impaired phenotype switching in the presence of oxidized lipoproteins. This impaired state is marked by increased production of pro-inflammatory cytokines and prothrombotic microparticles [7,8,9], along with enhanced surface expression of scavenger receptors [10,11]. When exposed to LDL oxidized by copper ions (oxLDL) or myeloperoxidase (Mox-LDL), THP-1-derived MΦs show increased lipid uptake, underscoring the central role of oxidized lipoproteins in foam cell formation [11,12]. Interestingly, despite the predominantly pro-inflammatory effects, oxLDL has been reported to drive human MΦ differentiation toward an anti-inflammatory M2-like phenotype [13]. These findings raise several important questions. If a Mox-like phenotype exists in human MΦs, would it share functional characteristics with murine Mox cells, such as reduced phagocytic and chemotactic capacity, or diverge in important ways? For instance, could human Mox-like MΦs be a hybrid phenotype, overlapping M1- and M2-like states, marked by the secretion of pro-inflammatory cytokines like TNF-α alongside chemotactic factors such as TGF-β1? If so, might they instead display enhanced chemotactic potential, contrary to observations in murine Mox cells?

Mox-like cells with heightened chemotactic activity could contribute to chronic inflammation in tissues by promoting cytokine-driven immune activation, while simultaneously recruiting additional immune cells, particularly mast cells. Such dual activity would likely intensify the inflammatory response within the arterial wall, ultimately leading to the development of atherosclerotic plaques. TGF-β1, a potent chemoattractant capable of inducing mast cell migration even at femtomolar concentrations [14,15,16], is produced by M2-like MΦs through SMAD2-dependent signaling pathways [17]. Clinically, mast cells are known to migrate from the adventitial to the intimal layer of the artery during atherogenesis, resulting in their colocalization with MΦs [18]. However, the underlying mechanisms driving this migration remain unclear. Direct interactions between these two cell types are shown to facilitate foam cell formation [19] and contribute to the development of vulnerable plaques [20,21]. As we previously demonstrated, oxLDL at a subclinical concentration (8 µg/mL) significantly enhances TNF-α secretion from resting state (M0) human MΦs and concurrently activates human mast cells, as evidenced by a 2.5-fold increase in histamine release [8]. TNF-α and histamine exert a synergistic detrimental effect on vascular endothelial cells, promoting monocyte-endothelial adhesion and facilitating monocyte recruitment and accumulation in the arterial intima [8,22]. These findings suggest that, in the presence of oxLDL within the intima, co-localized MΦs and mast cells become activated and induce vascular endothelial damage through extensive release of cytokines, thereby driving plaque formation.

Another important question is whether pro-atherogenic changes in human MΦs can occur even in the absence of extracellular LDL oxidation, driven instead by MΦ-secreted cytokines such as interleukin-6 (IL-6). Native LDL alone does not induce MΦ dysfunction unless it becomes aggregated by extracellular matrix proteoglycans [23,24]. However, native LDL internalized by MΦs can undergo intracellular oxidation as a result of IL-6–induced ROS overproduction and mitochondrial dysfunction [25,26,27], potentially leading to effects similar to those caused by oxLDL. IL-6 is known to influence MΦ polarization, e.g., it upregulates the expression of CD206, a marker of the M2-like cells, and increase the CD206/CD86 expression ratio, where CD86 is a M1-like state marker [28]. Furthermore, patients with a family history of premature coronary artery disease exhibit significantly elevated plasma IL-6 levels [29], underscoring the important role of this cytokine in atherogenesis.

Here, we investigate whether native LDL or its oxidized form can cause mast cell migration toward MΦs. Particularly, using ELISA and adhesion and migration assays, we test the hypothesis that oxLDL or LDL in combination with IL-6 polarizes human MΦs to a Mox-like state, intermediate between M1- and M2-like phenotypes, that not only secretes pro-inflammatory cytokines but also promotes TGF-β1-mediated mast cell migration.

## 2. Materials and Methods

### 2.1. Reagents

Human native LDL and its copper sulfate-oxidized form (oxLDL) as well as recombinant human TGF-β1, IL-4, IL-13, and IFN-γ were purchased from Thermo Fisher Scientific (Waltham, MA, USA). Recombinant human IL-6 and Phorbol 12-myristate 13-acetate (PMA) were acquired from Novus Biologicals (Centennial, CO, USA) and Millipore-Sigma (Burlington, MA, USA), respectively. Bacterial LPS powder was a gift from Dr. Granger (LSU Health Sciences Center at Shreveport).

### 2.2. Cell Culture

THP-1 human acute monocytic leukemia cell line was obtained from ATCC (Manassas, VA, USA). THP-1 cells were grown in RPMI 1640 (ATCC) medium supplemented with 10% fetal bovine serum (Thermo Fisher), 1% penicillin/streptomycin (Thermo Fisher), and 0.05 mM 2-mercaptoethanol (Millipore-Sigma). The LUVA human mast cell line was a gift from Dr. Steinke (University of Virginia). LUVA cells were grown in SFM medium with StemPro-34 supplement and 2 mM L-glutamine (Thermo Fisher). Primary human umbilical vein endothelial cells (HUVECs) were obtained from Thermo Fisher and cultured in Medium 200 supplemented with Low Serum Growth Supplement and Gentamicin/Amphericin B (Thermo Fisher).

### 2.3. Macrophage Differentiation

THP-1 monocytes (1.0 × 10^5^ cells/mL) were differentiated into M0 MΦs by applying 100 ng/mL PMA for 48 h. The polarization of M0 MΦs into M1-like or M2-like phenotypes was achieved by 48 h culture in the growth medium where PMA was replaced with 20 ng/mL IFN-γ + 100 ng/mL LPS or 20 ng/mL IL-4 + 20 ng/mL IL-13, respectively. M0, M1-like, and M2-like MΦs were incubated in the medium containing 20 ng/mL IL-6, 8 µg/mL LDL, 8 µg/mL oxLDL, or their combination for 24 h. The medium was then replaced with fresh growth medium, and in next 48 h, the conditioned medium was collected for the following experimental groups: MΦ + LDL, MΦ + oxLDL, MΦ + IL-6, MΦ + LDL + IL-6, and MΦ + oxLDL + IL-6.

### 2.4. Enzyme-Linked Immunosorbent Assay (ELISA) Measurement

TGF-β1 and TNF-α ELISA kits (Eagle Biosciences, Amherst, NH, USA) were used to measure the concentration of TGF-β1 released from MΦs in the above-described groups and TNF-α released from M0, M1-like, and M2-like MΦs. The MΦ conditioned media combined with 1N hydrochloric acid (HCl) were first placed in wells of a standard 96-well plate and stored in 2–8 °C for 1 h to activate TNF-α or TGF-β1 in the media. HCl was then neutralized by mixing with 1 N sodium hydroxide (NaOH). After the neutralizing procedure, the wells of a 96-well ELISA plate, pre-coated with TNF-α or TGF-β1 monoclonal antibody, were filled with 100 μL of either ELISA standards or experimental group samples for 1.5 h at 37 °C. These samples were then removed and a 100 μL solution of biotin-conjugate was added to each well and incubated for 1 h at 37 °C. After removing the biotin-conjugate, 100 μL of streptavidin-horse radish peroxidase was added to the well and incubated at 37 °C for 30 min. The last two steps were to replace streptavidin-horse radish peroxidase with the substrate solution (15 min incubation) and to replace the substrate solution with the stop solution. The TNF-α and TGF-β1 concentrations were measured from the light absorbance at the wavelength of 450 nm by a microplate reader.

### 2.5. Static Adhesion Assay

HUVECs (passage 4–5) were seeded into a 96-well plate at a density of 0.4 × 10^5^ cells/well and incubated to form an endothelial monolayer. At confluence, the cells were exposed to the conditioned medium from untreated or treated M0, M1-like, and M2-like MΦs for 4 h. This medium was then replaced with RPMI 1640 medium containing THP-1 cells, labeled with Vybrant DiO dye (Thermo Fisher), with a density of 0.5 × 10^6^ cells/mL. After a 25 min incubation, the THP-1 suspension was carefully aspirated, and the wells were washed three times by phosphate-buffered saline (PBS) to eliminate non-adherent THP-1 cells. The endothelial monolayer was visualized using a ×10 objective in an inverted epifluorescence microscope (Eclipse TiS, Nikon, Tokyo, Japan), and fluorescent images of firmly adherent THP-1 cells were taken by a digital CCD camera (Retiga EXi, QImaging, Surrey, BC, Canada) at three randomly selected fields. The field size was 905 µm in width by 675 µm in length. The recorded images were processed by custom data analysis software to determine the number of firmly adherent cells.

### 2.6. µ-Slide Chemotaxis Assay

LUVA mast cells were cultured in the 30 mm Petri dish and activated by 100 ng/mL PMA for 24 h, followed by application of cold PBS for cell detachment. Mast cells were then seeded in the center area of the µ-slide (Ibidi USA, Fitchburg, WI, USA). The cells in both the dish and the µ-slide were maintained at a concentration of 5.0 × 10^5^ cells/mL, following the recommended protocol [30]. The growth medium and chemoattractant agent including M0, M1, M2, M1 + LDL, M1 + IL-6, M1 + LDL + IL-6, M1 + oxLDL, M1 + oxLDL + IL-6, or 2 ng/mL TGF-β1 were applied to the right-wing part (sink) and left-wing part (source) of the slide, respectively. The trajectories of migrating cells were reconstructed from phase-contrast images taken in the viewing area of the central channel (2 mm × 1 mm) every 5 min for 12 h. Images were acquired by a CMOS camera (aca1920, Basler AG, Ahrensburg, Germany) connected to the Nikon Ti-S inverted microscope with a ×10 objective.

### 2.7. Data Analysis 

Images of migrating mast cells were analyzed by ImageJ (v. 1.42q, NIH, Bethesda, MD, USA) and “Chemotaxis and migration tools” (v.2, Ibidi USA). The root-mean-square displacement of the cells was calculated from displacements of individual cells, *d_i_*, *i* = 1,…, n, as follows:

(1) $$d_{\mathrm{RMS}}=\sqrt{\frac{1}{n}\sum_{i=1}^{n}d_{i}^{2}}$$

The directed migration of the cells was assessed by the forward migration index (FMI) calculated as the average ratio of the distance of the cell from its initial location to the end point, *d_i,c_*, to its path length, *l_i,c_*:

(2) $$\mathrm{FMI}=\frac{1}{n}\sum_{i=1}^{n}\frac{d_{i,c}}{l_{i,c}}$$

Three to six independent experiments per group were conducted. Statistical analysis of the experimental data was conducted using Prism software (v. 8.4.2, GraphPad, La Jolla, CA, USA). The data were presented as mean ± standard error of the mean (SEM). The *p*-values were calculated using one-way ANOVA and Tukey’s multiple comparisons test. A statistical significance threshold was set at *p* < 0.05.

## 3. Results

### 3.1. OxLDL Induces the Polarization of M0 MΦs Toward a Hybrid Mox-like Phenotype

**Figure 1:**
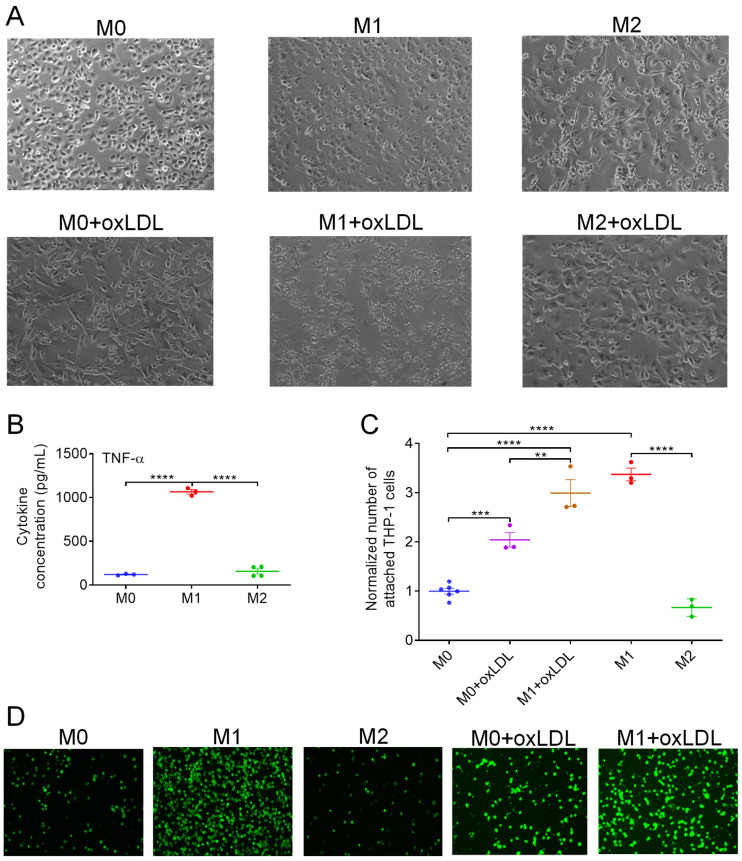
Brightfield images (**A**) of M0, M1-like, and M2-like MΦs treated or not by oxLDL. The concentration of TNF-α (**B**) released by M0, M1-like, and M2-like MΦs. The number (**C**) and fluorescent images (**D**) of THP-1 cells attached to HUVECs exposed to conditioned media from, respectively, untreated and oxLDL-treated M0, M1-like, and M2-like MΦs. The number of attached cells was normalized to the mean of the M0 group. Mean ± SEM of 3–6 independent experiments. ** *p* < 0.01, *** *p* < 0.001, **** *p* < 0.0001.

THP-1 cells differentiated into M0 MΦs by PMA treatment were adherent cells with a nearly round shape (Figure 1A, top left). MΦs retained a slightly elongated shape upon polarization to the M1-like state but began forming filopodia and became more dispersed from one another (Figure 1A, top middle). Polarization to the M2-like state resulted in clustered populations of highly elongated cells (Figure 1A, top right). These morphological changes were consistent with prior observations in polarized MΦs derived from THP-1 cells [31] and monocytes from patients with age-related macular degeneration [32]. The morphology of M0 MΦs treated with oxLDL resembled that of untreated M2-like cells (Figure 1A, bottom left). In M1-like cells exposed to oxLDL, clustering and filopodia/lamellipodia formation were more pronounced as compared to untreated M1-like MΦs (Figure 1A, bottom middle). M2-like MΦs treated with oxLDL exhibited morphology similar to that of untreated M2-like cells (Figure 1A, bottom right). These shape changes support that oxLDL induced a polarization shift in M0 and M1-like MΦs toward the M2-like state. As shown in our previous data (cf. Figure 2 in [8]), oxLDL-treated M0 MΦs exhibited a more than sixfold increase in TNF-α secretion compared to untreated M0 MΦs (771.8 vs. 120.0 pg/mL). This level was only slightly lower than that observed in M1-like MΦs (1065 pg/mL, Figure 1B). In contrast, M2-like MΦs secreted TNF-α at a concentration as low as untreated M0 MΦs (156.5 vs. 120.0 pg/mL, Figure 1B). These findings indicate that oxLDL induced a pro-inflammatory state in M0 MΦs, despite their morphology resembling that of anti-inflammatory M2-like cells. An alternative explanation is that oxLDL disrupts normal phenotype transitions in M0 MΦs, generating a mixed population of M1- and M2-like cells.

The pro-inflammatory phenotype of oxLDL-treated M0 and M1-like MΦs was further supported by THP-1 cell adhesion data. As shown in Figure 1C,D, endothelial cells exposed to conditioned medium from oxLDL-treated M0 MΦs exhibited a twofold increase in the number of firmly adherent THP-1 cells compared to those exposed to medium from untreated M0 MΦs (Figure 1C). The highest number of adherent cells was observed with medium from untreated M1-like MΦs, followed closely by oxLDL-treated M1-like MΦs (3.4- vs. 3.0-fold relative to the M0 level). In contrast, endothelial cells exposed to M2-like MΦs showed minimal THP-1 adhesion (0.67-fold of the M0 level, Figure 1C), consistent with their anti-inflammatory phenotype.

**Figure 2:**
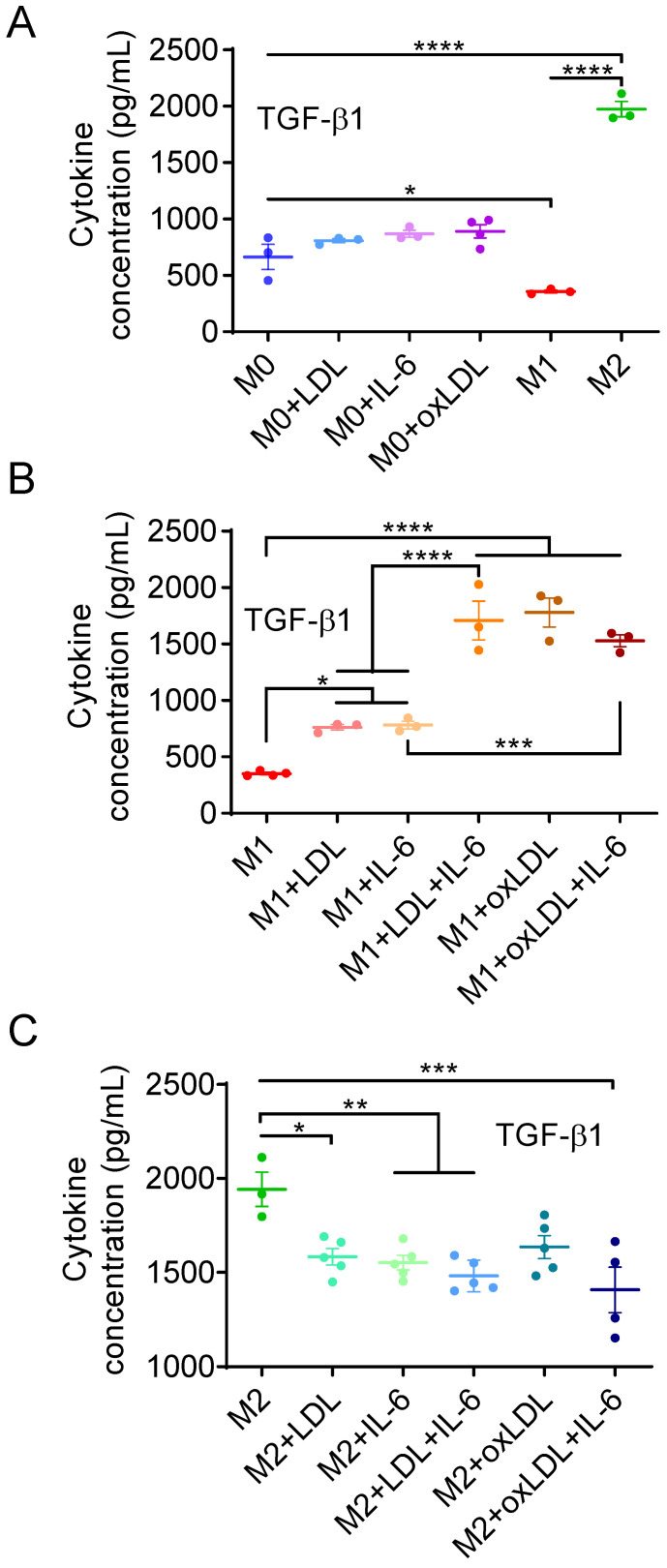
The concentration of TGF-β1 released by M0 (**A**), M1-like (**B**), and M2-like (**C**) MΦs treated or not by IL-6, LDL, oxLDL or their combination. Mean ± SEM of 3–5 independent experiments. * *p* < 0.05, ** *p* < 0.01, *** *p* < 0.001, **** *p* < 0.0001.

The concentration of TGF-β1, a marker of the M2-like state, was 1750 pg/mL in the conditioned medium of M2-like MΦs, which was 3.0 and 5.5 times higher than in the media from M0 (664.5 pg/mL) and M1-like (357.5 pg/mL) MΦs, respectively (Figure 2A). Interestingly, oxLDL stimulated M0 MΦs to secrete slightly elevated levels of TGF-β1 (891.5 pg/mL). Native LDL and IL-6 also increased TGF-β1 secretion, reaching 807.7 and 869.6 pg/mL, respectively (Figure 2A). Although these treatments did not significantly increase TGF-β1 relative to untreated M0 cells, they all produced significantly higher TGF-β1 levels (*p* < 0.05) in M0 MΦs compared with M1-like MΦs. This indicates that LDL, oxLDL, and IL-6 did not induce M1-like polarization but instead shifted resting cells toward a hybrid phenotype characterized by concurrent secretion of TNF-α and TGF-β1.

### 3.2. OxLDL or LDL in Combination with IL-6 Induces the Polarization of M1-like MΦs into a Mox-like Phenotype

Exposure of M1-like MΦs to IL-6 alone, LDL alone, LDL + IL-6, or oxLDL induced TGF-β1 secretion. Treatment with IL-6 or LDL alone resulted in a ~2.1-fold increase in TGF-β1 levels (from 352.0 to 762.0 and 781.3 pg/mL, respectively; Figure 2B). The increase in TGF-β1 concentration was more pronounced with LDL + IL-6 and oxLDL, reaching 1708 and 1779 pg/mL (4.8- and 5.0-fold increase), corresponding to 86% and 90% of the M2-like level (1942 pg/mL), respectively (Figure 2B). In contrast, TGF-β1 secretion by M2 MΦs slightly reduced upon treatment with IL-6, LDL, LDL + IL-6, or oxLDL to 1408–1635 pg/mL (73% to 84% of the untreated M2-like level, Figure 2C). These results indicate that most M1-like MΦs transitioned to a hybrid Mox-like phenotype when exposed to oxLDL or the combination of LDL and IL-6. 

### 3.3. Mast Cells Migrate Toward Mox-like MΦs

**Figure 3:**
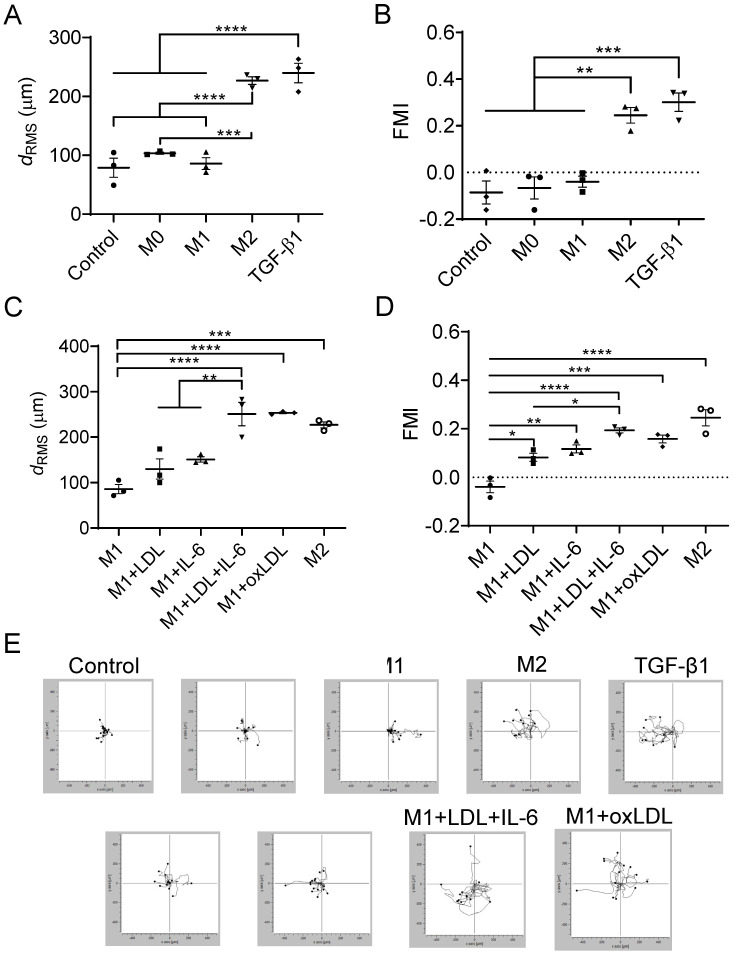
RMS displacement (**A**,**C**), forward migration index (**B**,**D**), and trajectories (**E**) of mast cells migrating in Ibidi chemotaxis channels in the control, M0, M1, M2, M1 + IL-6, M1 + LDL, M1 + LDL + IL-6, M1 + oxLDL, and TGF-β1 groups. The lines and black dots in panel (**E**) represent the trajectories and final positions of the cells, respectively. Mean ± SEM of 3 independent experiments. * *p* < 0.05, ** *p* < 0.01, *** *p* < 0.001, **** *p* < 0.0001.

Although TGF-β1 has been shown to induce chemotaxis of mouse mast cells [14], its effect on human mast cells remains less established. To address this, we investigated whether TGF-β1 or MΦ conditioned media could promote chemotaxis of LUVA human mast cells. As shown in Figure 3A, TGF-β1 significantly increased LUVA cell motility, as measured by root-mean-square (RMS) displacement (239.8 μm in the TGF-β1 group vs. 78.85 μm in the control group; *p* < 0.0001). The M2-like MΦ conditioned medium also enhanced motility (226.9 μm), which was slightly lower than that induced by TGF-β1 but significantly higher than in the M1 group (85.88 μm) (*p* < 0.0001).

Directional chemotactic migration was assessed using the forward migration index (FMI), which is positive when cells move toward a chemotactic source and negative when they move away. In representative cell trajectories (Figure 3E), the chemotactic source was positioned to the left of the LUVA cells. The highest FMI was observed in the TGF-β1 group (0.30), followed closely by the M2-like group (0.25), with no significant difference between them. In contrast, the control, M0, and M1 groups showed no chemotaxis, as indicated by negative FMI values: –0.08, –0.07, and –0.04, respectively.

Exposure of LUVA cells to conditioned media from M1-like MΦs treated with IL-6 or LDL slightly increased RMS displacement to 151.1 μm and 130.0 μm, respectively (Figure 3C,E). The cell motility was further and significantly enhanced when M1-like MΦs were treated with LDL + IL-6 or oxLDL, resulting in mast cell displacements that exceeded those induced by TGF-β1 (250.9 μm and 253.2 μm, respectively; *p* < 0.0001). Directional migration, as assessed by FMI data and cell trajectories (Figure 3D,E), progressively increased with MΦs treated with LDL alone (0.08), IL-6 alone (0.11), oxLDL (0.16), and LDL + IL-6 (0.20). All increases were statistically significant (*p* < 0.05 or less), and the FMI for the M1 + LDL + IL-6 group was not significantly different from that of the M2 group.

These findings indicate that Mox-like MΦs, polarized from M1-like cells by oxLDL and more notably by LDL combined with IL-6, exhibit enhanced chemotactic activity. Specifically, mast cell migration toward Mox-like MΦs was markedly greater than toward untreated M1-like MΦs and comparable to the response elicited by M2-like MΦs.

## 4. Discussion

MΦs play a central role in atherosclerosis. Their abnormal interactions with endothelial and tissue resident cells contribute to atherogenesis [33,34]. They differentiate into foam cells, which are a key hallmark of early atherosclerotic lesions [35]. In advanced stages, MΦs work in concert with mast cells to degrade the extracellular matrix, thereby contributing to the formation of vulnerable plaques, the primary cause of heart attacks and strokes [20,21]. The differentiation of blood monocytes into resting state (M0) MΦs after migrating through the endothelial lining, and their polarization into M1-like and M2-like phenotypes within the artery wall, are essential for maintaining tissue homeostasis [36,37]. However, MΦs are very sensitive to their environment and can adopt abnormal phenotypes when exposed to atherogenic stimuli [38].

Polarization into the Mox phenotype has been proposed as a contributing factor in human plaque development [38,39] based on previous studies using murine cells, which identified Mox as a dysfunctional MΦ phenotype that arises when resting-state MΦs are exposed to oxLDL [5]. However, the polarization to this phenotype was not demonstrated for human MΦs, which behavior can drastically differ from that of murine cells [40,41,42]. For example, the phenotypic profiles of pro-inflammatory (M1-like) and anti-inflammatory (M2-like) states in human MΦs do not mirror the distinct signatures observed in murine M1 and M2 MΦs [40]. Murine M1 MΦs prominently express inducible nitric oxide synthase (iNOS) and CD80 [43,44], whereas human M1-like MΦs express CD86 and exhibit markedly lower iNOS levels [45,46,47]. Both M2 and M2-like MΦs share the surface marker CD206 [42,44], but they differ in other marker expression: murine M2 cells express arginase-1 (Arg-1) [48], while human M2-like cells secrete transforming growth factor-beta1 (TGF-β1) [49,50]. Additionally, human MΦs often have overlapping phenotypes, characterized by the simultaneous expression of inflammatory and anti-inflammatory cytokines, as observed in human tumor-associated MΦs [41] and human microglia [51]. The different phenotypic profiles of polarized human and murine MΦs can, in part, be explained by differences in their metabolic responses. For example, human MΦs exhibit greater resistance to chemical stressors and, unlike their murine counterparts, do not undergo a metabolic shift from oxidative phosphorylation to aerobic glycolysis upon LPS stimulation [42]. Our findings indicate that, in the presence of oxLDL, human MΦs polarize into a Mox-like state that differs from the murine Mox phenotype by the simultaneous secretion of pro- and anti-inflammatory factors, such as TNF-α and TGF-β1, and an enhanced chemotactic capacity.

The polarization into the Mox-like state was particularly pronounced in M1-like MΦs, which are prevalent in the inflamed arterial walls due to the significantly higher local concentration of IFN-γ (M1-polarizing cytokine) compared to IL-4, the primary driver of M2 polarization [52]. This suggests that Mox-like cells can drive atherogenesis at the sites of tissue inflammation. Moreover, Mox-like polarization can be triggered not only by oxLDL but also by native LDL or the pleiotropic cytokine IL-6, with the effect becoming particularly pronounced (comparable to that of oxLDL) when MΦs are exposed to both LDL and IL-6 simultaneously. Our data point out that the Mox-like phenotype arises from a defective transition between the M1-like and M2-like state. When IL-6 initiates a shift toward the M2-like phenotype in the presence of LDL or oxLDL, this transition may be disrupted, resulting in polarization to a dysfunctional Mox-like state instead.

**Figure 4:**
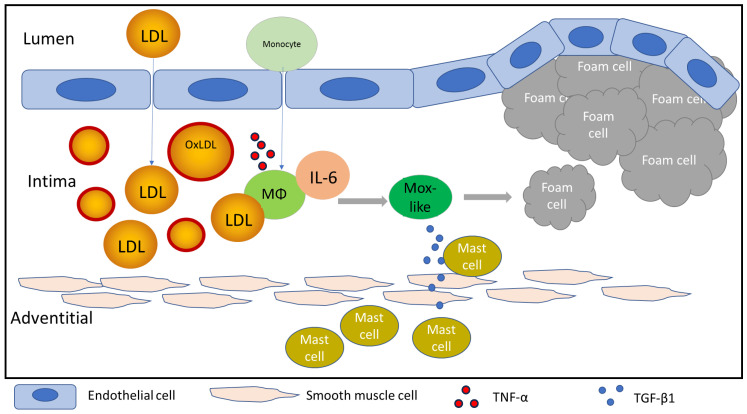
The schematic graph illustrating mast cells migration toward Mox-like MΦs and the potential transformation of Mox-like cells into foam cells in the intimal layer of the artery.

A key consequence of MΦ polarization to the Mox-like state is the recruitment of mast cells to the intimal layer of the artery. Mast cells are powerful immune cells rich in granules that contain fully active proteases and inflammatory mediators [53,54]. Upon activation or degranulation, they release this cargo to combat pathogens, induce tissue damage, and amplify the inflammatory response. Premature or excessive activation of mast cells results in severe pathophysiological conditions, such as anaphylaxis [55], and is implicated in various autoimmune disorders, including rheumatoid arthritis and multiple sclerosis [56]. Furthermore, mast cell overproduction (mastocytosis) significantly increases the risk of life-threatening allergic inflammation [57]. Mox-like cells produce TGF-β1, a cytokine involved in cell proliferation and collagen production [58], which also functions as a potent chemoattractant for mast cells [14,15,16]. Once co-localized with MΦs in the intima, mast cells can be activated or undergo degranulation in response to oxLDL [37]. The release of pro-inflammatory mediators from activated mast cells can promote foam cell formation [19], likely involving Mox-like MΦs (Figure 4). Although the mechanism underlying mast cell-MΦ co-localization in the intima has not been well characterized, our findings suggest that TGF-β1 secreted by Mox-like MΦs may drive this process.

In addition to its relevance to atherogenesis, this study may shed light on mechanisms underlying neurodegeneration and Alzheimer’s disease (AD). Microglia are the resident MΦ of the brain and share many functions with peripheral MΦs, including the regulation of both the induction and resolution of inflammation [59]. These dual functions are enabled by their ability to interconvert between M1 and M2 states [60]. In aged individuals, reduced integrity of the blood–brain barrier (BBB) [61], combined with nondestructive transcytosis across the BBB [62,63], may facilitate LDL accumulation within the brain, where it can subsequently be oxidized by astrocytes [64]. In a recent report on statin risk for dementia, the American Heart Association pointed out that the brain has a stable amount of LDL cholesterol [65]. Additionally, brain tissue contains substantial amounts of IL-6, produced by glial cells and neurons to regulate neuronal phenotype and function [66]. IL-6 overproduction in the brain can directly induce neurodegeneration [67], and this cytokine has been implicated as a potential contributor to AD-like symptoms in patients with long COVID [68,69]. Thus, microglia in the aged brain can potentially be exposed to LDL/oxLDL and IL-6 and may consequently adopt a Mox-like phenotype. It should also be noted that the brain-resident mast cells are preferentially located near the BBB in the hippocampus (first brain region affected during AD development) [70], where they function as a source of serotonin [71] and modulate anxiety-like behavior [72]. Importantly, mast cell numbers increase during AD progression as demonstrated in post-mortem analyses of AD patients [73]. Mast cell mediators such as tryptase and histamine have been shown to induce the pro-inflammatory activation of microglia [74,75], and the contribution of microglia–mast cell crosstalk to chronic neuroinflammation is now well established [76,77]. Supporting this concept, masitinib, an experimental drug that inhibits activation of both mast cells and microglia, has shown significant clinical benefit in individuals with mild-to-moderate AD in Phase III clinical trials [78]. Further studies are needed to determine whether microglia can adopt a Mox-like state in the aging brain and contribute to AD pathogenesis.

In addition to masitinib, the following therapeutic approaches can be used to prevent pathophysiological effects of Mox-like cells. The action of IL-6 can be blocked by anti-IL-6R antibody, e.g., tocilizumab or ALX-0061, which effectiveness was proven for autoimmune disease treatment [79,80]. The mast cell migration can be inhibited by blocking TβR-I and TβR-II, two receptors of TGF-β1 highly expressed in migrating mast cells [16]. The combination of these two therapeutic regimens would prevent inflammatory and wound healing responses in healthy tissues that cause atherogenesis. Our future work will focus on a comprehensive characterization of the Mox-like phenotype using flow-cytometry markers, targeted proteomics, and lipidomic analysis, as well as on inhibiting Mox-like polarization and its downstream effects through pharmacological blockers of IL-6 activity and mast-cell migration.

## Data Availability

The raw data supporting the conclusions of this article will be made available by the corresponding author on request.
